# Treatment of Peripheral Facial Paralysis After COVID-19 Infection With Traditional Chinese Medicine Therapies: A Case Report

**DOI:** 10.7759/cureus.57047

**Published:** 2024-03-27

**Authors:** Dong Li, Larissa Tao, Zihe Chen, Wa Cai, Weidong Shen

**Affiliations:** 1 Department of Acupuncture, Shuguang Hospital Affiliated to Shanghai University of Traditional Chinese Medicine, Shanghai, CHN

**Keywords:** case report, covid-19, peripheral facial palsy, traditional chinese medicine, acupuncture

## Abstract

Peripheral facial paralysis, characterized by facial expression and motor dysfunction of facial muscle groups, stems from lower motor neuron lesions of the facial nerve and can arise from various medical conditions such as viral infections, trauma, tumors, and autoimmune disorders, among others. It affects individuals across all age groups, from pediatric to geriatric populations. While many cases have no discernible cause, some are associated with infectious or non-infectious factors. Typically, most patients experience gradual recovery within one to three months following appropriate treatment in the acute phase, which may include inflammation control, antiviral therapy, reduction of neuroedema, and nerve nourishment. Although relatively rare, there have been few reports of peripheral facial paralysis following COVID-19 infection. Here, we present a case possibly linked to COVID-19: a 23-year-old male who reported numbness, facial asymmetry, and ear pain on the right side of his face persisting for five days after contracting COVID-19. Upon physical examination, peripheral facial paralysis of House-Brackmann grade IV was observed, prompting the initiation of traditional Chinese medicine (TCM) treatment. On the 10th day of treatment, acupoint catgut embedding was introduced as an adjunct therapy. Following four weeks of combined treatment, the patient's peripheral facial paralysis improved to grade I, and treatment was subsequently discontinued. TCM therapies, including acupuncture, electroacupuncture, plum blossom needle, moxibustion, acupoint catgut embedding, Chinese herbal medicine, etc., are safe and promising complementary treatments for the acute management of peripheral facial paralysis. However, additional large-scale, randomized controlled studies are needed to determine whether these interventions have a significant additive or synergistic effect on achieving full recovery in patients with peripheral facial paralysis.

## Introduction

Peripheral facial palsy poses a challenge to many physicians across different medical specialties. It can be caused by several conditions, including but not limited to idiopathic peripheral facial palsy, herpes simplex, herpes zoster, trauma, diabetes, pregnancy, neoplastic infiltrates, and other rare conditions. The link between herpes simplex virus-1 (HSV-1) and peripheral facial palsy has been studied since the 1970s [1]. However, in recent years, a few cases of peripheral facial palsy have been reported following COVID-19 infection or vaccine injection [2,3]. This article presents a case study of a patient who suffered right facial paralysis following a COVID-19 infection.

This article will be presented as a meeting abstract at the Society for Academic Research/Research Center for Management and Innovation (SAR/RCMI) PolyU Conference 2024 on May 23, 2024.

## Case presentation

Initial treatment

**Figure 1:**
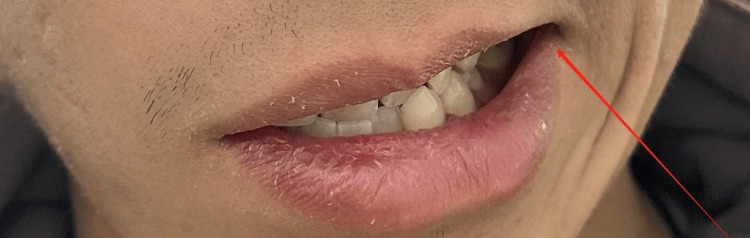
During initial evaluation: asymmetry of mouth with maximal effort (right)

**Figure 2:**
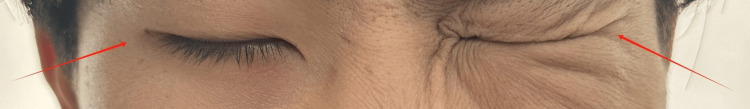
During initial evaluation: eyelid insufficiency (right); complete, strong eye closure (left)

**Figure 3:**
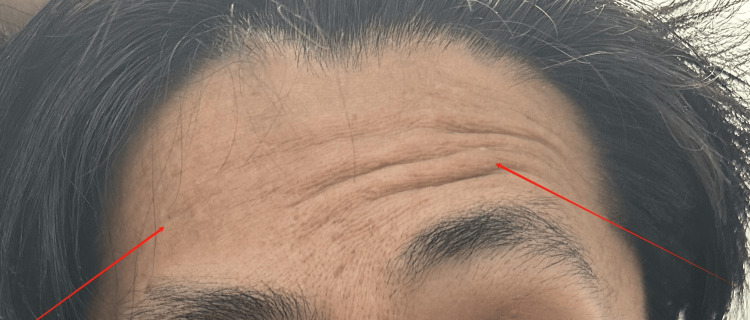
During initial evaluation: inability to lift brow (right)

**Figure 4:**
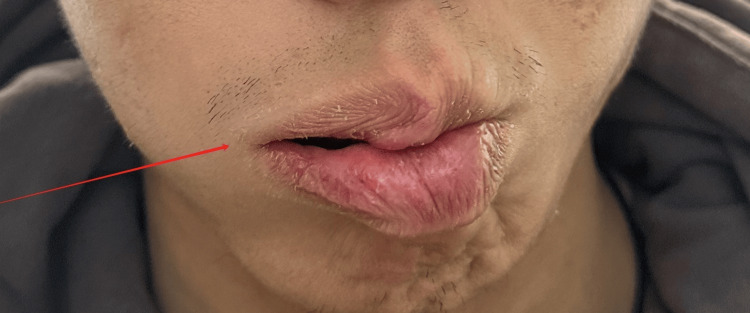
During initial evaluation: air leakage when puffing (right)

The patient was infected with COVID-19 on January 1, 2023, presenting with fever, aversion to cold, cough, headache, and other symptoms, and was treated with ibuprofen. After taking a shower and being exposed to wind on January 5, 2023, the patient had numbness, deviation, and ear pain on the right side of the face the next day. Since the patient's COVID-19 antigen test remained positive, he was self-isolated without medical treatment, and thus, his facial symptoms gradually aggravated. On January 11, 2023, the patient tested negative for COVID-19 antigen and had no symptoms of COVID-19 infection such as fever and aversion to cold. The patient came to our department to treat peripheral facial paralysis. Both of his parents and his three younger brothers were in good health. He denied any history of peripheral facial paralysis in his family and refuted the presence of infectious diseases like hepatitis, as well as familial hereditary conditions such as hypertension and diabetes among his relatives. Physical examination showed stiffness and numbness on the right side of the face, lagophthalmos of the right eye with tears, deviation on the right side of the mouth, inability to raise the right eyebrow, ability to keep food between the right cheek, water leakage from the right mouth when gargling, air leakage from the right cheek when puffing, pain in the right ear, and loss of taste and numbness on the front 2/3 part of the right side of the tongue. The appearance of the right external auditory canal and the right tympanic membrane was normal. There were no hearing loss, speech impairment, tongue extension deviation, or other abnormalities. The House-Brackmann score was IV (Figures 1-4).

The patient had normal appetite, bowel movements, and urination, but reported poor sleep quality. The tongue was pale with a white coating, and the pulse was floating and tense. The diagnosis was indicative of facial paralysis with a syndrome characterized by wind-cold-phlegm obstruction. Treatment aimed to alleviate symptoms by dispersing wind, expelling cold, eliminating phlegm, and dredging the collaterals. Differential diagnosis includes (1) The absence of the right frontal crease, coupled with the lack of hearing loss, speech impairment, and tongue extension deviation, led to the temporary exclusion of central facial paralysis, as brain CT and MRI examinations were declined by the patient. (2) No blisters were observed on the patient's right ear or other facial areas, and there was no inflammation or changes in hearing. Due to the patient's refusal of biopsy and blood tests, Hunt syndrome could not be diagnosed at this time.

The treatment prescriptions were as follows: (1) Acupuncture: shallow needling at Yin Xiang (LI20) (right), Si Bai (ST2) (right), Di Cang (ST4) (right), Jia Che (ST6) (right), Xia Guan (ST7) (right), Yang Bai (GB14) (right), Quan Liao (SI18) (right), Yi Feng (SJ17) (right), Cheng Jiang (RN24), Yu Yao (EX-HN4), Tai Yang (EX-HN5) (right); direct needling at He Gu (LI4) (bilateral), Tai Chong (LR3) (bilateral). Retaining needles for 30 minutes each time, once a day. (2) The oral herbal medicine prescription was modified Qianzheng powder and Daotan decoction, including Pinellia tuber 15g, Poria 12g, Coix seed 12g, Arisaema with bile 9G, tangerine reel 9G, bitter orange 9G, white silkworm 6G, divaricate Saposhnikovia root 6G, scorpion 3G, centipede 3G, white aconite root 3G, liquorice root 3G, decocted in 150ml water, 13once in the morning and evening, after meals.

Second treatment

**Figure 5:**
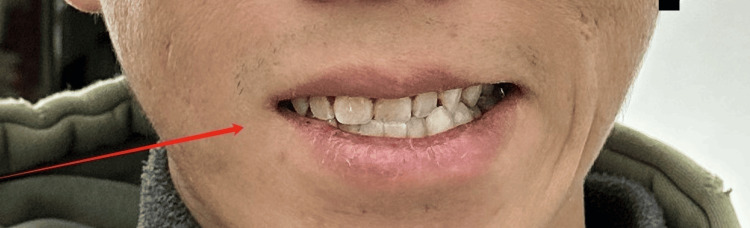
During mid-treatment evaluation: asymmetry of mouth with maximal effort (right)

**Figure 6:**
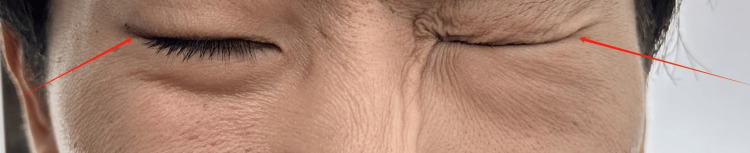
During mid-treatment evaluation: eyelid insufficiency (right); complete, strong eye closure (left)

**Figure 7:**
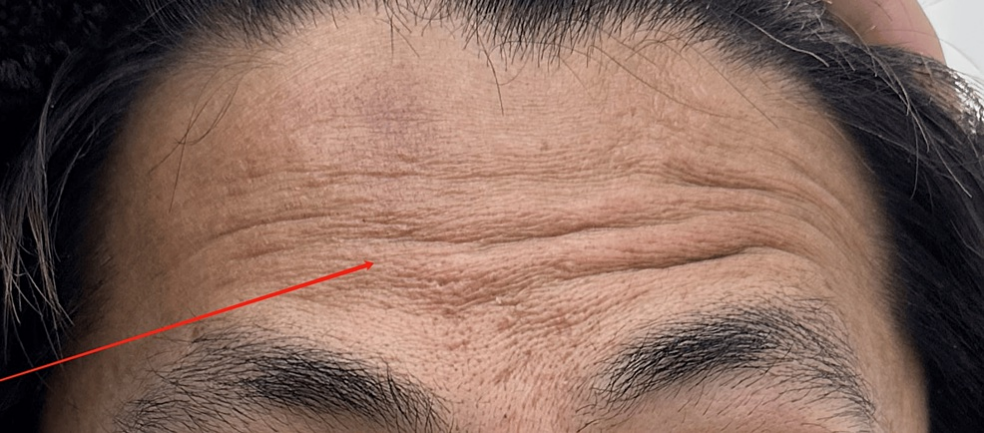
During mid-treatment evaluation: inability to lift brow (right)

**Figure 8:**
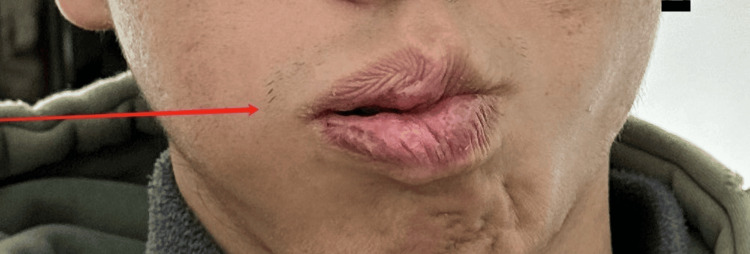
During mid-treatment evaluation: air leakage when puffing (right)

The above methods were employed continuously for a duration of four days. During the initial two days, the patient's symptoms deteriorated gradually, which was attributed to the nature of the acute phase of the disease. The doctor advised the patient to continue with the treatment. Beginning on the third day of treatment, the patient's symptoms began to stabilize, and there was no longer any pain behind the right ear (Figures 5-8).

The tongue was light red with a thin and white coating, and the pulse was stringy. As a result, the treatment prescriptions were modified as follows: (1) Acupuncture and electroacupuncture: the acupuncturist perpendicular inserted with 0.25mm diameter*40mm length needles at Yin Xiang (LI20) (right), Si Bai (ST2) (right), Tai Yang (EX-HN5) (right), Xia Guan (ST7) (right), Yang Bai (GB14) (right), Cheng Jiang (RN24), Yi Feng (SJ17) (right), Quan Liao (SI18) (right), He Gu (LI4) (bilateral), Tai Chong (LR3) (bilateral); transverse inserted with 0.25mm diameter*75mm length needles at Cuan Zhu (BL2) (right) towards Yu Yao (EX-HN4), and at Di Cang (ST4) (right) towards Jia Che (ST6) (right). After the patient felt the needling sensation (“deqi”), the acupuncturist connected two groups of acupoints with an electroacupuncture stimulator (SDZ-II, Huatuo Company, Suzhou, China) (Yang Bai (GB14) (right) and Tai Yang (EX-HN5) (right), Quan Liao (SI18) (right) and Xia Guan (ST7) (right)), selected continuous wave, set the amount of current according to the patient's tolerability. All needles remained in situ for 30 minutes. Disposable sterile stainless steel acupuncture needles were used for all treatments. (2) Plum blossom needle: the tip of the plum blossom needle was polished with sandpaper and sterilized. After disinfection of the skin, plum blossom needles were perpendicularly tapped on the diseased part of the face, and stopped when the skin turned slightly red or the patient experienced a burning sensation. There should be fewer bleeding points. Cotton balls were applied on the insertion areas to prevent subcutaneous blood stasis, once a day. (3) Moxibustion: moxibustion at Yi Feng (SJ17), moxibustion, and acupuncture were performed simultaneously, 30 minutes each time, once a day. (4) The oral herbal medicine prescription: Chinese Angelica root 9g and earthworm 6g were added to the previous prescription. Other dosages remained the same as before.

The above methods were employed continuously for a duration of five days.

Third treatment

The patient reported that the symptoms of loss of taste and numbness in the tongue improved gradually, but consistent treatment was challenging during the Chinese New Year period. Furthermore, the patient desired a faster recovery. The patient's tongue was pale and dark with a thin and white coating, and the pulse was stringy. Therefore, acupoint catgut embedding was added to the treatment plan.

The specific procedure was as follows: the acupoints selected were Yang Bai (GB14) (right), Tai Yang (EX-HN5) (right), Quan Liao (SI18) (right), Di Cang (ST4) (right), Jia Che (ST6) (right), and Zu San Li (ST36) (bilateral). For safety precautions and surgical preparations, the skin was disinfected with iodopovidone, surgical sterile gloves were worn, and a section of 4-0 PGA wire was taken using tweezers and placed at the front end of the #7 thread embedding needle. The needle was tilted at a 15° angle for facial acupoints and vertically for limb acupoints. Once it was ensured that the thread had entered the body and elicited the needling sensation (“deqi”), the needle was removed. The patient was advised not to wash their face or take a bath on that day and to keep the wound clean and dry. Thread embedding therapy was administered once every seven days, for a total of two times. The oral prescription was switched to Modified Taohong Siwu Decoction: Chinese Angelica root 12g, Szechuan lovage rhizome 9G, red peony root 9G, Platycodon root 9G, earthworm 9G, tangerine peel 9G, Kudzuvine root 6G, peach seed 6G, safflower 6G, and liquorice root 3G, decocted in water, once in the morning and evening. The remaining treatments remained unchanged from the previous course.

Outcome

**Figure 9:**
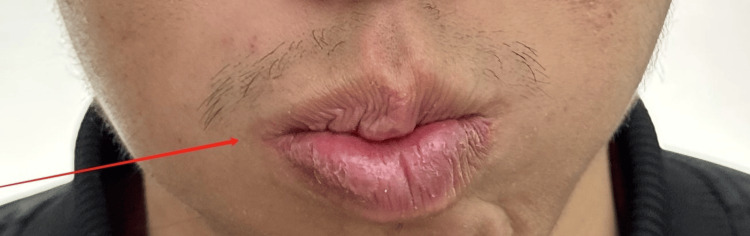
During post-treatment evaluation: symmetry of mouth when whistling

**Figure 10:**
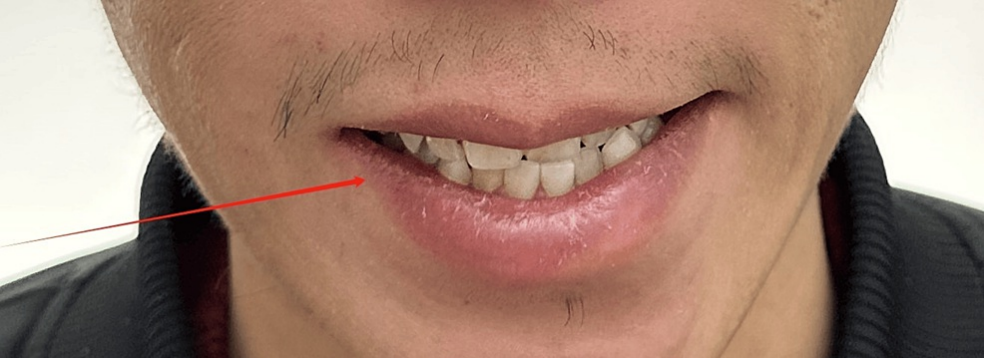
During post-treatment evaluation: showing teeth and puffing

**Figure 11:**
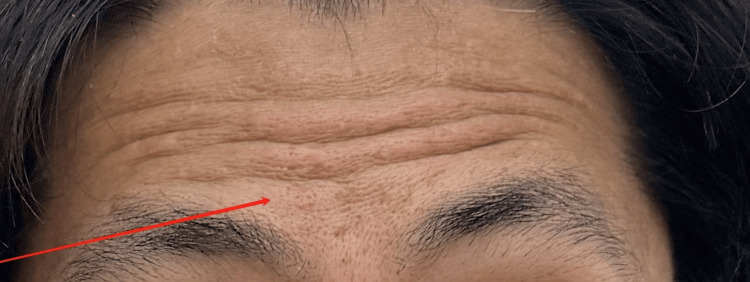
During post-treatment evaluation: symmetrical forehead lines when raising eyebrows

**Figure 12:**
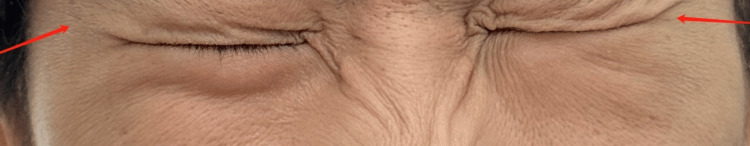
During post-treatment evaluation: normal eye closure

Following four weeks of treatment, the patient's facial numbness and mouth and eye deflection symptoms had significantly improved. The bilateral forehead lines were nearly symmetrical, the right eyelid could be lifted, and the eyes could be closed normally. The patient was able to eat without difficulty, there was no leakage while gargling, and the facial muscles and nasolabial groove had almost returned to normal. The House-Brackmann rating was Grade I (Figures 9-12).

Furthermore, the patient had a good appetite, normal sleep patterns, and normal stool and urine. The tongue was light red with a thin and white coating, and the pulse was stringy.

## Discussion

Understanding of this disease

Peripheral facial palsy caused by a virus is a common and acute disease in clinics, which is mainly manifested by facial expression muscle group dysfunction, and even accompanied by abnormal taste, hearing, and other symptoms. The incidence rate is 11-40 individuals per 100,000 each year [4]. The pathogenesis of this disease currently includes inflammation theory [5], virus infection theory [6], microcirculation disorder theory [7], etc. This case is considered a facial nerve injury caused by COVID-19 and improper nursing after infection.

The outlook for peripheral facial palsy suggests that around 70% of patients experience a complete and spontaneous recovery, but for 15~20%, there may be minor cosmetic sequelae, while the remainder face moderate to severe consequences that can lead to dissatisfaction with the outcome. Facial muscle dysfunction and numbness can be debilitating, affecting patients' lives and having a significant impact on their psychological and social well-being, as well as their overall quality of life. Most patients receive reasonable treatment in the acute phase, and can gradually recover or recover within one to three months, but those who delay for more than one year and still have no signs of recovery have a poor prognosis [8]. Given the negative impact of these conditions, it is crucial to have safe and effective treatment options that can increase the chances of complete recovery without any sequelae.

While there is currently no conclusive evidence linking peripheral facial paralysis to either COVID-19 infection or vaccination, there have been reports and studies worldwide that suggest a possible correlation between the two since the onset of the COVID-19 pandemic [2]. Additionally, adverse events of facial paralysis have been reported in individuals who have received mRNA COVID-19 vaccines [9]. In fact, a study published in The Lancet Infectious Diseases demonstrated that the risk of developing Bell's palsy appears to be elevated following vaccination with coronavirus vaccines such as BNT162b2 and CoronaVac [10]. Since the epidemic of COVID-19, the number of people infected with COVID-19 has increased significantly, and a large number of cases related to COVID-19 sequelae have also occurred (such as facial paralysis, hair loss, memory decline, etc.). Combined with the concept of long-term COVID [11], considering that this patient suffered from a cold during COVID-19 infection, it is speculated that his facial paralysis might be related to COVID-19 infection, and the correlation needs further research.

Western medicine mainly treats the disease with antiviral drugs such as acyclovir, nutritional nerve drugs such as mecobalamin, glucocorticoids such as prednisone, or intravenous injection of dehydrating agents such as mannitol to reduce nerve edema, intramuscular injection of fasudil to improve microcirculation, intramuscular injection of rat nerve growth factor to promote nerve growth and recovery, ginkgo biloba extract antioxidant and free radical scavenging treatment [12,13]. Hyperbaric oxygen and rehabilitation therapy can be used as supplementary therapy [14,15]. The patient declined treatment with Western medicine, and thus the doctor refrained from employing these therapies.

Treatment of this case

The doctor holds that, initially, the patient's body was attacked by the wind and cold epidemic virus, leading to the depletion of his vital energy and weakness of the body. Following a cold shower during recovery, the pathogens entered through the skin, obstructing the flow of qi and blood in the channels on the face. This, in turn, resulted in the development of facial paralysis, which is commonly recognized as the syndrome of wind and phlegm blocking the collaterals. It should be treated by taking into account both deficiency and excess, dispelling wind, resolving phlegm, and dredging collaterals. Use mild stimulation in the acute phase, and then gradually increase the amount of stimulation.

Reason for acupoints selection

Facial paralysis is linked to the six-yang meridians of the hands and feet. Di Cang (ST4) and Jia Che (ST6) are acupoints located on the Stomach Meridian of Foot Yangming, with Di Cang (ST4) intersecting with the Large Intestine Meridian of Hand Yangming and the Yang Heel Vessel. These acupoints are commonly used to treat facial diseases by dredging the facial meridians. Puncturing Di Cang (ST4) to Jia Che (ST6) may speed up the recovery of the damaged facial nerve [16]. Tai Yang (EX-HN5) and Yang Bai (GB14) can regulate facial meridians, warm them, disperse cold, and nourish facial muscles. Si Bai (ST2) is an acupoint on the Stomach Meridian of Foot Yangming, with the zygomatic branch of the facial nerve on its superficial layer and a nerve penetrating the infraorbital foramen on its deep layer. Cheng Jiang (RN24) is the intersection point of the Ren Meridian, the Large Intestine Meridian of Hand Yangming, the Stomach Meridian of Foot Yangming, and the Du Meridian, with the inferior alveolar nerve's terminal branch below it. Xia Guan (ST7) is the intersection point of the Stomach Meridian of Foot Yangming and the Gall Bladder Meridian of Foot Shaoyang, with several nerve branches in its superficial layer. Yin Xiang (LI20) is the intersection point of the Large Intestine Meridian of Hand Yangming and the Stomach Meridian of Foot Yangming, with the buccal branch of the facial nerve in its deep layer. Quan Liao (SI18) is the intersection point of the Triple Energizer Meridian of Hand Shaoyang and the Small Intestine Meridian of Hand Taiyang, with the zygomatic and buccal branches of the facial nerve on its superficial layer and the mandibular nerve branches of the trigeminal nerve on its deep layer. Yu Yao (EX-HN4) and Tai Yang (EX-HN5) are extra points with branches of the facial nerve, including the zygomatic-facial branch, the temporal branch of the facial nerve, and the temporal nerve of the mandibular nerve on the Tai Yang (EX-HN5) and branches of the facial nerve on the Yu Yao (EX-HN4). Si Bai (ST2), Cheng Jiang (RN24), Xia Guan (ST7), Ying Xiang (LI20), Quan Liao (SI18), Yu Yao(EX-HN4), and Tai Yang (EX-HN5) all have the function of unblocking qi and blood in the facial meridians and are commonly used acupoints for treating facial paralysis.

Regarding body acupoints, He Gu (LI4) is the primary point of the Large Intestine Meridian of Hand Yangming, and the focal point where the meridian energy accumulates. It is also a commonly used non-facial acupoint for treating facial diseases, as it can stimulate the Large Intestine Meridian of Hand Yangming and dredge the qi and blood of the meridians. Tai Chong (LR3) is situated on the Liver Meridian of Foot Jueyin, which runs through the face. It corresponds with He Gu (LI4), which is referred to as the "opening siguan acupoint" and can effectively dispel wind and clear blockages in the collaterals. Yi Feng (SJ17) belongs to the Triple Energizer Meridian of Hand Shaoyang, which can alleviate symptoms of wind and cold. It is an essential acupoint frequently used to treat wind-related diseases and facial paralysis, with the facial nerves passing through it extensively. The deep layer of Yi Feng (SJ17) is where the facial nerve trunk enters from the stem-mastoid hole and is accompanied by multiple blood vessels.

Methods of acupuncture and moxibustion

Facial paralysis has different invasion depths at different stages. In the early stage of facial paralysis, external pathogens attack the surface, and most of the pathogens are on the surface of the body. Pathogens enter the depth from the surface during the quiescent period. In the recovery period, the pathogen enters deeper into the meridians. Experts suggest that different treatment methods should be taken for this disease at different stages. In the acute phase, patients should pay attention to rest and reduce adverse stimulation, and doctors' treatments are mainly to protect the nerves. During the recovery period, doctors moderately stimulate the facial nerve, promote nerve regeneration, and repair nerves’ function [17].

Therefore, in this case, facial acupoints were only slightly punctured in the acute phase. Once the patient's condition stabilized, the doctor employed methods such as electroacupuncture, penetrating needling, and plum blossom needle, which generated a considerable amount of stimulation. The acupuncture needles were also inserted more profoundly into the skin.

Moxibustion could unblock channels and activate collaterals, dispel cold, and dissipate phlegm. Moxibustion in the acute phase could strengthen the body's positive qi and disperse pathogens on the surface. In the middle and late stages of the disease, moxibustion could dredge the qi and blood of the meridians.

Application of Chinese herbal medicine

The syndrome types of this condition change throughout different stages, and therefore, Chinese herbal medicine should be modified to correspond with the specific syndrome.

In the acute phase, the patient's tongue was pale, the fur was white, and the pulse was floating and tense, which was the syndrome of wind-cold-phlegm obstruction. The treatment was to dispel wind, cold, phlegm, and dredge collaterals. The oral prescription was Modified Qianzheng Powder and Daotan Decoction. The prescription consisted of Pinellia tuber, Poria, Coix seed, Arisaema with bile, tangerine reel, bitter orange, white silkworm, divaricate Saposhnikovia root, scorpion, centipede, white aconite root, and liquorice root. Arisaema with bile could eliminate dampness and phlegm, dispel wind, and disperse knots; white aconite root could dispel wind and phlegm; bitter orange could promote Qi and eliminate phlegm, all of which were monarch drugs. Scorpion, white silkworm, and centipede could dispel wind and dredge collaterals, Pinellia tuber could dry dampness and dispel phlegm, tangerine reel could promote qi and eliminate phlegm, divaricate Saposhnikovia root could dispel the wind and relieve the external symptoms, all of which were minister drugs. Poria and Coix seed are assistant drugs for strengthening the spleen and removing dampness. Liquorice root was a guide drug that played a role in harmonizing. In the second treatment, the symptoms of facial paralysis tended to be stable and entered the static period. Combined with tongue and pulse, Chinese Angelica root and earthworm were added to strengthen the function of promoting qi, activating blood circulation, and unblocking collaterals. In the third treatment, the patient's symptoms improved day by day. Combined with tongue and pulse, traditional Chinese medicine (TCM) was changed to Modified Taohong Siwu Decoction. Chinese Angelica root promoted blood circulation without hurting blood. Red peony root, Szechuan lovage rhizome, peach seed, and safflower cooperated with Chinese Angelica root to promote blood circulation and remove blood stasis. The earthworm could activate the meridians and collaterals, which could spread all over the body, and activate blood without damaging the vital-qi. Kudzuvine Root introduced various drugs into the Yangming meridian, and Platycodon root carried drugs up. Tangerine peel could promote qi and eliminate phlegm, Liquorice root could harmonize. The whole prescription achieved the purpose of unblocking qi and activating blood circulation.

Application of acupoint catgut embedding therapy

Acupoint catgut embedding therapy means implanting absorbable protein sutures into the acupoints and continuously stimulating the meridians and acupoints. This therapy has been widely applied in the treatment of peripheral facial paralysis [18]. The therapy was advantageous due to its extended treatment duration and sustained acupoint stimulation, which could decrease the frequency of patient visits. It was especially useful for patients who could not regularly see a doctor but needed prompt and effective treatment. Given that the patient expressed some fear due to their first experience with acupoint catgut embedding therapy, the doctor utilized fewer but more precise acupoints. This approach aimed to minimize excessive stimulation and observe the patient's tolerance to this treatment for future adjustments.

Limitations

Due to economic considerations and the patient's preferences, we refrained from conducting blood routine tests, electromyography, and other assistant examinations. Clinical outcomes were assessed using the House-Brackmann grading system and the patient's subjective reports. However, it's crucial to incorporate assessments of both functional improvements and aesthetic outcomes when evaluating the effectiveness of the treatment. Due to the absence of standardized protocols for acupuncture and other therapies, it is challenging to recommend these interventions for individuals experiencing peripheral facial palsy following a COVID-19 infection. It was difficult to determine the individual contributions of acupuncture and other therapies to the patient’s recovery. Further clarification is needed on the etiology, differential diagnosis, and treatment plan of this disease.

## Conclusions

As epidemic prevention policies change and COVID-19 continues to evolve, the number of COVID-19 sequelae is bound to increase significantly. Peripheral facial paralysis can greatly impact patients' quality of life, so early intervention is crucial to reduce the duration of the disease and improve treatment outcomes. TCM therapies are safe and promising complementary options for the acute management of peripheral facial paralysis, playing important roles in the treatment process. However, further large-scale, randomized controlled studies are necessary to assess whether these interventions have significant additive or synergistic effects in achieving complete recovery for patients with peripheral facial paralysis.

## Appendices

Patient perspective

After I was infected with COVID-19 one month ago, I took a bath when the virus was positive. The next day, I had facial paralysis. In the first week of the disease, the virus test was still positive. I had to isolate at home, and the facial paralysis became more and more serious. My family told me that acupuncture could treat facial paralysis. At first, I was very afraid of needles and received treatment with fear. After four weeks of treatment, I felt almost normal. I was very satisfied with these treatments. However, I hope that you will have the ability to reduce the pain of acupuncture in the future.
